# Scaling-up malaria treatment: a review of the performance of different providers

**DOI:** 10.1186/1475-2875-11-414

**Published:** 2012-12-12

**Authors:** Mohga M Kamal-Yanni, Julien Potet, Philippa M Saunders

**Affiliations:** 1 Campaign and Policy Division, Oxfam, John Smith Drive, Oxford, OX4 2JY, UK; 2 International Consultant, Paris, France; 3 International Consultant, London, UK

**Keywords:** Malaria, Community health workers, Private sector, Public sector, Treatment, ACT

## Abstract

**Background:**

Despite great progress towards malaria control, the disease continues to be a major public health problem in many developing countries, especially for poor women and children in remote areas. Resistance to artemisinin combination therapy (ACT) emerged in East Asia. Its spread would threaten the only effective malaria treatment currently available. Improvement in availability of diagnosis as part of malaria control has highlighted the fact that many fevers are not due to malaria. These fevers also need to be promptly diagnosed and adequately treated in order to improve public health outcomes in developing countries.

**Methods:**

This review looked for evidence for the most effective approach to deliver malaria treatment in developing countries, by public sector, formal and informal private sector, and community health workers (CHWs). The authors analysed 31 studies to assess providers based on six parameters: knowledge and practice of provider, diagnosis, referral practices, price of medicine, availability of ACT, and treatment coverage and impact on morbidity and mortality.

**Results:**

The public sector has made progress in prevention and treatment in many countries, but facilities are inaccessible to some communities, and the sector suffers shortages of health workers and stock-outs of medicines. Despite wide outreach, the private sector, especially informal facilities, presents public health risks. This is due to an inability to diagnose and treat non-malarial fevers, and an innate motive to over-prescribe malaria treatment. The need to pay for treatment is a major factor in deterring poor women and children from accessing the medicines they need. A system that depends on ability to pay risks a repeat of the chloroquine story, where an effective and cheap anti-malarial drug was rendered useless partly due to under-treatment. CHWs have proved to be effective agents in providing correct diagnosis and treatment of malaria and other common fevers, even in remote areas.

**Conclusions:**

The evidence shows that there is no short-cut to investing in training and supervision of providers, or in treating malaria within a public health context rather than as a separate disease. The studies highlighted that all outlets face challenges in delivering their services, but that CHWs scored highly in almost all parameters. CHWs have proved to be effective agents in providing correct diagnosis and treatment of malaria and other common fevers, even in remote areas. Their role should be recognized and expanded.

## Background

Malaria treatment is at a crossroads. While the numbers of cases and deaths have decreased due to successful prevention and treatment, the emergence of resistance to artemisinin combination therapy (ACT) threatens to halt progress towards malaria control and elimination. In particular, the emergence of parasite resistance in areas such as the Greater Mekong region sounds alarm bells for the efficacy of ACT in the medium term. If resistance to ACT is to be contained, rational use of the treatment must be a global priority.

Scaled-up prevention and effective treatment programs are key to malaria control and, ultimately, elimination. There are a number of prerequisites for effective use of ACT. These include the provider’s knowledge of, and adherence to, treatment protocols; the abandonment of ineffective and sub-standard medicines and monotherapies; the provision of education for care-givers and patients; and access to ACT by all those who need treatment. In addition, correct diagnosis with rapid diagnostic tests (RDTs) or microscopy, recommended by the WHO, is essential to successful health outcomes given that the concordance rates between ‘presumptive’ and ‘actual’ parasitological malaria cases amount to between 10% and 60% depending on the season, the age of patients, and the transmission area [1,2]. Presumptive treatment can lead to many non-malarial fevers being treated incorrectly with costly ACT, endangering the patient’s life and wasting precious household, national, and donors’ resources on useless treatment.

A number of national programmes have succeeded in combining effective diagnosis with treatment. Countries such as Ethiopia, Zambia, and Rwanda have managed to achieve impressive results, and have reduced mortality and morbidity by scaling up prevention, diagnosis, and treatment delivered through the public sector and by community health workers (CHWs) [3-5].

Since 2004, delivery strategies for ACT have focused on the Affordable Medicine Facility for malaria (AMFm), a subsidy scheme that aims to increase access to ACT by making medicines more affordable in the private sector [6]. The AMFm pilot evaluation showed increased sales and reduced prices in the private sector, but there was no data on treatment of actual malaria [7]. In November 2012, the Global Fund, which hosts the AMFm, decided to integrate the AMFm into its normal grant structure, thus leaving the decision on financing types of providers to individual countries [8].

The debate about treatment providers tends to focus on public and private sectors. This study is based on a literature review of available evidence on the effectiveness of different delivery modes for ACT through public, private (formal and informal), and CHWs’ systems. It is hoped that the findings will help countries to decide on the most effective routes to invest aid and domestic finances to achieve universal coverage of ACT for confirmed malaria patients.

## Methods

### Selection criteria

The criteria for the inclusion of studies were: 1) a location where ACT was a first-line treatment for uncomplicated malaria at the time of the study; 2) high-prevalence countries in sub-Saharan Africa and Cambodia, where resistance to ACT has already started; 3) studies with defined provider(s); and 5) a clearly defined methodology.

All the studies were published in English or French in peer-reviewed journals, except for three studies that were included because they presented relevant findings that had been the subject of very little investigation in studies in peer-reviewed journals. The review excluded opinion pieces and publications on the implementation of the AMFm, in anticipation of the evaluation of the AMFm pilot programme.

### Types of studies

Included studies were of two main types: observational and intervention-related studies. Most observational studies compared the current performances of some providers in rolling out ACT. Most intervention-based studies were pre-/post-intervention studies, focusing mainly on a single intervention or policy.

### Search strategy and information sources

The health-care literature was searched through PubMed using the key words ‘malaria’, ‘malaria drug therapy’, ‘ACT’, and ‘anti-malarials’ as a major subject. These keywords were used in combination with terms referring to different types of provider: ‘public health facilities’; ‘public sector’; ‘community health workers’; ‘community medicine distributors’; ‘malaria volunteers’; ‘part one pharmacies’; ‘drug shops’; ‘private medicine retailers’; ‘patent medicine dealers’; ‘wholesalers’; ‘self-medication’; ‘over the counter’. Reference lists of relevant publications, including systematic reviews, were also hand-searched.

### Selection of studies

Applying the inclusion criteria resulted in pre-selection of 105 studies. These were read to select studies that included data on the performance of treatment providers. Eventually 31 studies were considered to provide relevant data and thus were analysed (listed in Table 1).

**Table 1:** List of studies and investigated performance

**No**	**Study**	**Country**	**Provider**	**Performance studies**
1	Abdelgader *et al*. [26]	Sudan	Public health facilities	1, 2, 4
2	Ajayi *et al*. [14]	Ghana, Uganda, Nigeria	Community medicine distributors (CMDs)	1, 6
3	Amuasi *et al*. [9]	Burundi	Public, private	1, 4, 5
4	Batwala *et al*. [33]	Uganda	Public	1, 2, 4
5	Batwala *et al*. [17]	Uganda	Public health centres	2
6	Bhattarai *et al*. [6]	Zanzibar	Public health-care facilities	4
7	Chanda *et al*. [5]	Zambia	CHWs	1, 2, 3
8	Chinbuah et al. [15]	Ghana	CHWs	1, 2, 3
9	Cohen *et al*. [51]	Tanzania	Small drug shops	4, 5, 6
10	Counihan *et al*. [27]	Zambia	CHWs	2
11	Ewing *et al*. [29]	Malawi	CHWs	2, 3, 5, 6
12	Kamat & Nyato [36]	Tanzania	Public health-care facilities	5
13	Kisia *et al*. [13]	Kenya	CHWs	6
14	Lemma *et al*. [3]	Ethiopia	CHWs	2, 3
15	Littrell *et al*. [48]	Cambodia	Public and private outlets, formal and informal	2, 4, 5
16	Littrell *et al*. [20]	Benin, DRC, Madagascar, Nigeria, Uganda, Zambia	Formal and informal private sector	4
17	MSF publication [38]	Mali	Public health-care facilities/CHWs	4, 5, 6
18	O’Connell *et al*. [10]	Benin, DRC, Nigeria Madagascar, Uganda, Zambia	Public, private formal and informal	4, 5
19	Onwujekwe *et al*. [30]	Nigeria	Public health-care facilities/formal and informal private facilities	1, 2, 4, 6
20	Rusk *et al*. [12]	Kenya	Private outlets, formal and informal	1
21	Rutebemberwa *et al*. [37]	Uganda	Public and formal/informal private sectors	3, 5
22	Sabot *et al*. [13]	Kenya, Senegal, Tanzania, Cambodia	Private facilities: formal and informal	1, 2, 4, 5
23	Sabot *et al*. [13]	Tanzania	Small drug shops	4, 5, 6
24	Thomson *et al*. [31]	Sierra Leone	CHWs	3
25	Wasunna *et al*. [43]	Kenya	Public health-care facilities	1, 2, 4
26	Wasunna *et al*. [44]	Kenya	Public health-care facilities	1, 4
27	World Bank [50]	Zambia	CHWs	4
28	Yasuoka *et al*. [16]	Cambodia	CHWs	1, 2
29	Yeboah-Antwi *et al*. [54]	Zambia	Community health workers	1, 2
30	Yeung *et al*. [28]	Cambodia	CHWs	1, 2, 4, 5
31	Yeung *et al*. [47]	Cambodia	Private	4, 5

1. Provider's knowledge and practice; 2. Diagnosis; 3. Referral; 4. ACT vs other malaria medicines; 5. Affordability; 6 Treatment coverage.

### Limitations

The review had several limitations: the studies used different methodologies and covered different data categories. Some studies focused on one provider while others covered several. Also, some countries were covered by a small number of studies, e.g. Mali, thus providing limited data compared with more extensively studied countries, e.g. Uganda.

## Results

**Figure 1:**
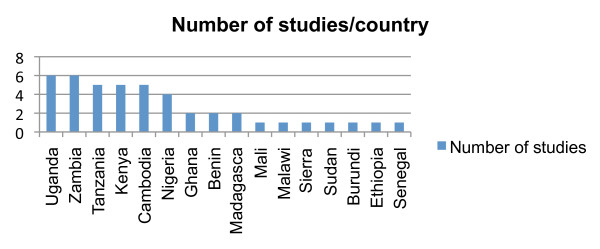
**Number of studies per country.** Some studies covered several countries.

The studies were located in 15 countries in sub-Saharan Africa and in Cambodia. Uganda and Zambia had the highest number of studies while seven countries were included in one study only (Figure 1).

### Types of provider

**Figure 2:**
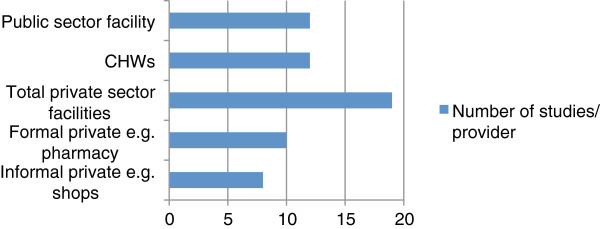
**Number of studies per provider.** Some studies covered several providers. One study did not specify whether the private sector was formal or informal, so it was added to the total private sector.

Four major types of provider were defined: public health facilities, community health workers, and formal and informal private outlets (Figure 2). In some studies, NGOs were involved as trainers and programme managers for private and CHWs programmes rather than as direct providers. The formal private sector includes facilities staffed by trained personnel, such as pharmacies, clinics, and regulated drug shops. The informal private sector covers a wide range of outlets: small drug shops with little or no regulation, general stores selling anti-malarials among other household commodities, and hawkers.

### Parameters of performance

Provider performance was categorized across six parameters, which were also used in analysing data from the studies. These were:

1. Knowledge and practices of provider: knowledge of and compliance with national malaria treatment guidelines and education of patients on doses and adherence to treatment;

2. Diagnosis: availability and use of diagnostics, compliance with test results;

3. Referral practices: prompt referral to a more qualified provider when necessary, e.g. in cases of severe malaria and negative RDTs;

4. Availability and use of ACT: availability, effective prescription of ACT, and ACT’s share by volume related to other anti-malarials;

5. Affordability: cost of medicines;

6. Treatment coverage, including for poor people.

**Figure 3:**
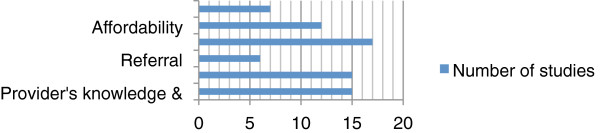
Number of studies per performance parameter.

Seventeen studies reported on ACT availability and 15 studies investigated providers’ knowledge, practice, and diagnosis, but only six studies reported on referral practices. This variation in coverage had an effect on the amount of data available on providers’ performance (Figure 3).

### Knowledge and practices of providers

Providers’ correct knowledge of doses, especially for children, and of the importance of adherence to the treatment course are essential for effective treatment. The studies showed great variations between providers, with CHWs consistently demonstrating retention and use of knowledge and good practice.

A survey in Burundi found that 96% of a public sector sample was familiar with the indication of ACT use, compared with 39% in the private sector [9]. However, in some countries, such as DRC, knowledge of malaria treatment was low in all sectors [10].

Informal private medicine retailers performed much worse in many settings. Only one in ten in a Nigerian study could correctly state the recommended first-line treatment for uncomplicated malaria, as opposed to one in four providers from formal pharmacies and a little less than one in two public providers [11]. Even where retailers were aware of treatment guidelines, and stocked ACT in their shops, few adhered to the recommendations. In a study in Kenya, only 48% of retailers would recommend ACT to adults and only 37% to children [12].

Studies of the formal private sector produced mixed results. A study in Tanzania found that drug dispensers in private pharmacies had poor knowledge of treatment guidelines, despite selling ACT [13]. However, a household survey in Kenya found that nurses from private outpatient clinics were more compliant with national treatment guidelines than workers from public facilities [13].

CHWs scored well in their knowledge and practices of treatment provision in most studies. In a multi-centred study in Ghana, Nigeria, and Uganda, an average 85 per cent of children receiving ACT from ‘community medicine distributors’ (CMDs) were correctly treated [14]. This was confirmed by a study in Ghana, where trained CHWs provided correct doses of ACT and also systematically observed the intake of the first dose. More than half of children’s care-givers came back to CHWs two to three days after administering drugs, as recommended [15]. In Zambia and Cambodia, services (delivery and explanation of correct doses) provided by CHWs were evaluated as positive [5,16].

### Diagnosis

WHO guidelines on malaria treatment state that treatment should be based on test results. Microscopy-based diagnostic capacities are supposed to be available within public health centres at intermediary level, but this is not the case in some developing countries. A study of referral units in rural Uganda found that only a minority had effective laboratories. As a result, only 9% of patients were examined for parasites [17]. However, microscopy and/or RDT were widely available in the public sector in Zambia, Madagascar, Cambodia, and DRC [18].

The availability of malaria diagnostics was very low among private outlets, except in Cambodia, Zambia, and Uganda, and almost none of the informal for-profit private outlets offered malaria diagnostics [19]. ACT watch surveys in six countries revealed that “children treated in the private sector are less likely to receive appropriate diagnostic testing and treatment as compared with children treated in the public sector” even though “the public sector was far from ideal” [20].

In general, studies showed that a high percentage of providers prescribed anti-malarials on the basis of positive test results [21]. However, some studies raised concerns that there was evidence that health workers prescribed ACT even for negative tests. For example, studies in Tanzania, Ghana, and Zambia showed that around 50% of febrile patients with negative malaria tests in the public sector were prescribed anti-malarials [22,23]. However, other studies documented higher levels of health workers adhering to test results and not prescribing ACT to patients with negative results [24]. This is illustrated by studies in the public sector in mainland Tanzania where over-treatment was only 20.9%, in Uganda (23.4%), and in Sudan (17%) [21,25,26].

All studies pointed to the fact that CHWs were the best providers in terms of ability to use RDTs, and to adhere to the test results [27]. They showed substantially lower rates of over-prescription of anti-malarials, and in some cases were also able to target the use of antibiotics. Two studies in Zambia found that CHWs were compliant with test results, leading to a dramatic decline in the number of unnecessary treatments with anti-malarials, reaching zero% in one study [5]. A study in Ethiopia confirmed these findings, that CHWs use RDTs effectively in better targeting positive patients [3].

In Cambodia, where testing and compliance are of particular importance due to rising resistance to artemisinin, CHWs have demonstrated good case management. For example, in the areas covered by ‘village malaria workers’ (VMWs) in rural Cambodia, individuals were 11 times more likely to receive a confirmed diagnosis than in areas where people used services from the private sector [28]. VMWs were able to restrict anti-malarials to those who had positive RDT results [28].

### Referral practices

Referral practices depended on a number of factors: whether care providers identified clinical reasons for referring, whether they properly advised patients on the need for referral, and whether patients were able to overcome cost and accessibility barriers. Only six studies reported on referral practices, yet they provide some indicators about providers’ performance on this parameter.

A study in Malawi in ‘hard-to-reach’ villages found that 89% of individuals who sought assistance from CHWs did not proceed to attend a formal health facility, despite CHWs being taught to refer febrile children [29]. Another study in Ghana found that only five out of 17 children with warning signs were referred to a health facility. CHWs argued that, even when referred, most care-givers would not go to the health facility because of the expense [15].

Limited data is available on referral practices by informal private retailers. However, a survey measuring the satisfaction of patients and care-givers in Nigeria showed that people were least satisfied with the follow-up service of ‘patent medicine dealers’ and pharmacy shops. One reason for this was that people were not properly counselled on what to do if their health did not improve after therapy [30].

Referral is particularly important in negative cases. A Médecins sans Frontières (MSF) study in Sierra Leone showed a generally low referral completion rate but more patients referred with severe malaria than with negative tests [31].

### Availability and use of ACT versus ineffective anti-malarials

Comparative surveys by ACT watch highlighted the persistence of non-recommended anti-malarials in private drug stores. In all countries except Cambodia, the private sector distributed substantially fewer doses of ACT than other anti-malarials. In DRC and Nigeria, oral artemisinin monotherapies (AMT) were found in as many as 50% of private outlets [19]. This was confirmed by a study in Burundi, where recommended ACT was available in 87% of public and 33% of private retail outlets, but non-recommended anti-malarials – including halofantrine, which is known for its dangerous side-effects and cross-resistance patterns with lumefantrine – were found more frequently in private outlets (39%) compared with public facilities (4%) [9]. However, chloroquine and sulphadoxine-pyrimethamine (SP) were no longer available in any outlet after they were banned nationally. This suggests that banning ineffective medicines could be an effective way for governments to stop the use of undesirable anti-malarials and monotherapy.

Surveys in Senegal and Madagascar showed that poor-quality medicines were not limited to any particular type of distributor. In Uganda, the public sector performed best, while in Nigeria, a country with weak drug regulations, oral AMT accounted for 10% of prescriptions in the public sector [30,32]. There was no significant difference in stocking ineffective medicines between the regulated and the informal private sector in those countries. This raises the question of whether the private sector (professional or informal) would stop selling ineffective medicines without a government ban.

Availability of ACT in the public sector is an important determinant of case management. [33]. Fear of stock-outs was an important factor in health workers ‘rationing’ ACT, reserving it for cases seen as ‘priority’ [25].

### Affordability

Most public sector facilities provided ACT free of charge, although some charged consultation fees [10]. In the private sector the price of ACT, if not subsidized, was much higher than that of ineffective anti-malarials such as chloroquine or SP. A survey in Burundi found that private sector medicine sellers were reluctant to simply stock artesunate-amodiaquine (ASAQ) as they were not able to match the price in the public sector [9].

Subsidizing ACT in the private sector (non-AMFm) demonstrated mixed results and challenges. Subsidy and social marketing in Madagascar reduced the price of ACT [18]. However, although private outlets in Senegal appeared to apply reasonable mark-ups on subsidized ASAQ, the retail price remained higher than that of SP. This could explain the fact that the availability of ASAQ hardly increased in private shops and that SP was still available in almost all private shops [34].

Two studies in Tanzania looked at subsidizing ACT at the top of the supply chain, to be sold to the local main drug wholesaler who, in turn, committed to sell those drugs only to specialized drug shops. The shops kept the retail price at a similar level to that of SP. A significant increase in the proportion of customers purchasing ACT was observed in the intervention districts, from 1% at baseline to 44% one year later compared with no increase in the control district [13]. Yet the Tanzanian experience showed that ACT was stocked more often in shops located closer to district towns and major roads and used more by individuals of higher socio-economic status. Shops in very remote areas were much less likely to stock subsidized ACT [13].

ACT subsidy in the public sector could take the form of the removal of user fees and provision of free medicines. According to the WHO, ACT is available free of charge for all age-groups in the public sector in 52 out of 77 countries where it is used for the treatment of *Plasmodium falciparum*[35]. Mothers interviewed in urban Tanzania said that the introduction of highly subsidized ACT in public health centres significantly reduced the cost of treating a malarial episode [36]. However, despite there being no official user fees at government facilities, in a sample in rural Uganda two-thirds of children with fever were taken to drug shops as the first source of care outside the home. The authors concluded that removing user fees did not necessarily make health care affordable, as other costs persist, such as cost of transport and loss of working time [37].

### Treatment coverage

The studies showed that wider treatment coverage was achieved by a combination of increased use of CHWs and free services. Two multi-centre studies demonstrated high treatment rates after community medicine distributors were deployed with free or highly subsidized ACT in Ghana, Nigeria, and Uganda. Household surveys found that between 52% and 75% of febrile children were reported to have received ACT from a CMD [14]. A study on the deployment of 33 CHWs with ACT in the under-served region of Tigray in Ethiopia showed that CHWs treated more than 75,000 people over two years. In the intervention districts, more patients were treated with ACT than in the non-intervention districts, and the intervention significantly lowered the risk of malaria-specific mortality – by approximately 40% during a two-year period [3].

CHWs in an MSF project in Mali distributed 35% of all paediatric ACT, mostly to remote populations in the rainy season, and thus reduced the proportion of severe malaria cases in primary health facilities from 6% to less than 2% [38].

A study in Zanzibar found that the introduction of free ACT in the public sector in 2003 was the main factor behind big decreases in infant and crude child mortality attributed to malaria: 75% and 71% respectively between 2002 and 2005. The number of children under five seeking care from public facilities increased two-fold after the introduction of free ACT [39].

### Analysis

This review covered 31 studies of different outlets that provided malaria diagnosis and treatment: public services, private services, and CHWs. The studies were investigated for evidence of performance by different providers across six parameters: knowledge and practices; diagnosis; referral practices; availability and use of ACT; affordability; and treatment coverage.

The studies highlighted that all outlets face challenges in delivering their services, but that CHWs scored highly in almost all parameters. With the right training and supervision, CHWs can be effective agents in providing correct diagnosis and treatment of malaria, as well as other common childhood illnesses [40]. On the whole, CHWs retained and effectively used knowledge about diagnosis, treatment, and referral.

The level of knowledge was better in the public sector than with private providers, although there was room for improvement in the former. There was strong evidence that the current level of knowledge of malaria treatment guidelines among informal private retailers is very low in most of the settings [41]. Even where knowledge has improved, there were high levels of inappropriate treatment of fever [42].

Although knowledge did not necessarily translate to adherence to treatment guidelines and correct advice, CHWs consistently performed better than other providers. Public sector workers rationed the use of ACT, saving it for severe cases because of fears of stock-outs. Retailers dispensed ACT on the basis of customer requests and their ability to pay [43].

The characteristics of providers’ training were not described in most studies and it was, therefore, difficult to draw conclusions about the impact of types of training on prescribing. A study in Kenya analysed the prescribing practices of rural health professionals before and after in-service training and found hardly any change in the proportion of febrile children treated with ACT, or in the workers’ education practices [44]. In-depth interviews with providers found that the concomitant introduction of RDTs had actually led to confusing messages about the use of ACT [43]. This case highlights the importance of appropriate provider-oriented training and supervision.

RDT**s** are becoming more widely available in developing countries, making it more feasible to comply with the WHO treatment guidelines to provide ACT only after testing. However, cost and correct actions based on the test results, especially dealing with negative results, remain critical challenges. CHWs provided free testing and were more likely to act on RDT results than other providers. In comparison with private medicine retailers, qualified public providers generally performed better, but a significant proportion did not comply with test results. However, there are indications that the percentage of providers treating negative cases is decreasing. Practices are deeply rooted following decades of presumptive treatment with chloroquine and SP. Relevant training and supervision is necessary to deliver effective care, irrespective of the nature of the provider.

Despite the evidence of the superior competence of trained CHWs in using RDTs and ACT, the emphasis continues to be on scaling up RDTs in the private sector [45]. This is despite potential public health risks of the use of ACT in informal private outlets, including inadequate regulation, the potential for over-prescribing to maximize profit, and inability to address non-malarial fevers. A study in six countries highlighted the importance of regulation and quality control to ensure effective case management [46].

Even the experience of Cambodia, which has been implementing social marketing managed by NGOs in the private sector for nearly a decade, has not shown that increased knowledge leads to an increase in the correct use of ACT, based on confirmed diagnosis, in the private sector. A study in the country highlighted the need for intense communication/education, support, and monitoring of programmes [47]. Another study, also in Cambodia, confirmed these findings and added that despite the popularity of the private sector, with good availability of medicines, the sale of medicine cocktails that did not in fact contain any anti-malarials was prevalent. The use of RDTs did not improve quality of treatment [48].

Many studies identified two factors as being essential to successful prescribing and use of RDTs: sufficient training and supervision [39]. Well-designed and implemented training with follow-up support was seen as an effective way to change even deeply rooted clinical practices. These are important interventions that need to be well planned and implemented whether the provider is public, an NGO, CHWs, or the private sector, including shops.

An essential aspect of malaria control is the ability to refer severe malaria and negative results to health facilities. While providing correct diagnosis and treatment in the community by trained CHWs may contribute to a decreased need for referral as illness is caught and treated early, better diagnosis may lead to increased need for referral, e.g. for children with severe pneumonia. Therefore, it is important to deal with malaria in the context of community health, designing strategies that enable diagnosis and treatment of common illnesses, including malaria, rather than focusing on treating malaria only.

Availability of anti-malarials was generally higher in the private sector than in the public sector. Given that treatment was offered for ‘presumptive malaria’, the size of the market is not a success indicator for malaria control.

In the public sector, fear of stock-outs caused workers to ration the use of ACT to save medicines for the most serious cases, even when the RDT was positive [49]. Improving supply chains for the public sector will therefore not only increase the availability of ACT in health centres, but also convince providers to *systematically* prescribe ACT for uncomplicated malaria. The World Bank supported a programme in Zambia which reinforced health districts by only one additional commodity planner responsible for helping facilities to order directly from the central level. This intervention led to impressive results: the availability rate of paediatric ACT reached 88%, in comparison with 51% in the control districts [50].

Non-AMFm studies on subsidizing ACT highlighted the equity issue, in that poor people and people in remote areas were less likely to benefit from the subsidy. The authors suggested that distribution of ACT through CHWs might have better results in reaching remote communities [51].

An MSF study in rural Mali provides useful insights into the factors that increase the use of services. Free RDTs and ACT in the public sector led to a 20% increase in the number of malaria cases treated with ACT. Greater use occurred only when user fees for all febrile children and pregnant women were removed, and CHWs were deployed [38]. The study indicated that subsidizing ACT may not be a strong enough incentive to attract a large proportion of poor children and pregnant women with fever into health facilities. Free treatment, comprehensive care for febrile conditions, and expanded use of CHWs are key factors in increasing access to and use of malaria treatment.

## Discussion

Effective malaria control requires a comprehensive strategy of prevention, correct diagnosis, and treatment. The focus on malaria in the past decade has resulted in two different strategies. The first, adopted by the AMFm, favours subsidies for ACT and now RDTs, with sales through private outlets. The second is the provision of free malaria services through strengthened networks of public providers and CHWs.

Promoting subsidized medicines in the private sector involves a number of formidable challenges: guaranteeing a nationwide affordable price, reaching poor people in remote areas, and ensuring accurate treatment. A single subsidy at the top of the distribution chain may have a less pronounced effect in remote areas in terms of availability and affordability in private outlets [52]. This is a major concern as remote communities are precisely the priority target of such policies, because of their limited access to other sources of appropriate anti-malarials.

Introducing diagnostics for private retailers to ensure correct treatment poses additional problems. Shopkeepers, even more than public providers, may be tempted to ignore negative malaria test results, as their profits are determined by the sale of medicines. Patients may also demand ACT when they have paid for diagnosis, even when the result is negative. Other medicines may be sold, even though a private shop is not qualified to diagnose other causes of fever or to treat them appropriately.

The designers of the private sector option believed that retailers were the preferred choice for distribution of ACT, because of ease of access, reliable drug supply, familiarity of staff with customers, and flexible times of opening [20]. However, CHWs could offer a successful alternative route to the unregulated private sector and can also fill the public service gap in delivering diagnosis and treatment near the patient, thus overcoming physical and financial barriers to treatment. CHWs are usually members of the community themselves and therefore understand beliefs, culture, and the socio-economic status of patients. There is also evidence that utilisation of CHWs is higher in remote areas, areas of small hamlets, and by poor and the poorest people [53]. There is clear evidence that CHWs correctly use RDTs, dispense ACT, and counsel patients, increasing treatment coverage in under-served areas in high and low seasons. Studies show a reduction in delays in seeking care after CHWs were deployed [15]. In addition, CHWs were able to manage the logistics of the drug supply, ensuring that no drug was expired or out of stock [16]. Moreover, CHWs equipped with malaria RDTs were able also to perform effective case management of child pneumonia [54].

Despite the evidence of the ability of trained CHWs to achieve positive health outcomes in terms of diagnosis, treatment, and referral, and in dealing with negative cases, there is currently no serious attempt to globalize investment in CHWs as a strategy to combat malaria.

## Conclusions

Malaria continues to be a public health threat in many developing countries, especially for poor women and children in remote areas. This review aimed to look for evidence for the most effective approach to deliver malaria treatment in developing countries by public and private sectors and by CHWs.

The studies show that there is no shortcut to investing in training and supervision of providers and in treating malaria within a public health context, rather than as a separate disease. CHWs have proved to be effective agents in providing correct diagnosis and treatment of malaria and other common fevers, even in remote areas.

## Competing interests

No conflict of interest.

## Authors’ contributions

MKY: designed and managed the research project, performed data collection and analysis of 11 studies, reviewed the data and analysis of the 31 studies, drafted and finalized the article. JP: performed data collection and analysis of 31 studies. PS: contributed comments to the design and implementation of the research project and contributed to the article writing. All authors read and approved the final manuscript.
